# *Trichodysplasia spinulosa*-Associated Polyomavirus Uses a Displaced Binding Site on VP1 to Engage Sialylated Glycolipids

**DOI:** 10.1371/journal.ppat.1005112

**Published:** 2015-08-24

**Authors:** Luisa J. Ströh, Gretchen V. Gee, Bärbel S. Blaum, Aisling S. Dugan, Mariet C. W. Feltkamp, Walter J. Atwood, Thilo Stehle

**Affiliations:** 1 Interfaculty Institute of Biochemistry, University of Tübingen, Tübingen, Germany; 2 Department of Molecular Biology, Cell Biology and Biochemistry, Brown University, Providence, Rhode Island, United States of America; 3 Department of Natural Sciences, Assumption College, Worcester, Massachusetts, United States of America; 4 Department of Medical Microbiology, Leiden University Medical Center, Leiden, The Netherlands; 5 Department of Pediatrics, Vanderbilt University School of Medicine, Nashville, Tennessee, United States of America; Tufts University School of Medicine, UNITED STATES

## Abstract

*Trichodysplasia spinulosa*-associated Polyomavirus (TSPyV) was isolated from a patient suffering from *trichodysplasia spinulosa*, a skin disease that can appear in severely immunocompromised patients. While TSPyV is one of the five members of the polyomavirus family that are directly linked to a human disease, details about molecular recognition events, the viral entry pathway, and intracellular trafficking events during TSPyV infection remain unknown. Here we have used a structure-function approach to shed light on the first steps of TSPyV infection. We established by cell binding and pseudovirus infection studies that TSPyV interacts with sialic acids during attachment and/or entry. Subsequently, we solved high-resolution X-ray structures of the major capsid protein VP1 of TSPyV in complex with three different glycans, the branched GM1 glycan, and the linear trisaccharides α2,3- and α2,6-sialyllactose. The terminal sialic acid of all three glycans is engaged in a unique binding site on TSPyV VP1, which is positioned about 18 Å from established sialic acid binding sites of other polyomaviruses. Structure-based mutagenesis of sialic acid-binding residues leads to reduction in cell attachment and pseudovirus infection, demonstrating the physiological relevance of the TSPyV VP1-glycan interaction. Furthermore, treatments of cells with inhibitors of N-, O-linked glycosylation, and glycosphingolipid synthesis suggest that glycolipids play an important role during TSPyV infection. Our findings elucidate the first molecular recognition events of cellular infection with TSPyV and demonstrate that receptor recognition by polyomaviruses is highly variable not only in interactions with sialic acid itself, but also in the location of the binding site.

## Author Summary

Viruses engage receptors on their host cell to initiate entry and infection. Members within a virus family are known to target different tissues and hosts and exploit different pathogenic mechanisms due to critical changes in receptor specificity. The human *Trichodysplasia spinulosa*-associated Polyomavirus (TSPyV) is known to cause a rare skin disease in immunocompromised individuals. The pathogenic mechanism includes hyperproliferation of inner root sheath cells, but molecular determinants underlying the infection and the associated disease remain unknown. Here we applied a structural and functional approach to investigate the recognition events during early infection steps of the virus. We found that TSPyV engages sialic acid receptors but employs a novel binding site on the capsid that is shifted in comparison to other structurally characterized polyomaviruses. Cell-based studies demonstrate the relevance of the observed interaction for attachment and infection and suggest that glycolipids, rather than N- and O-linked glycoproteins, are important for infection. Our findings demonstrate exemplarily that receptor recognition by (polyoma-) viruses is highly variable not only in interactions with sialic acids, but also in the location of the binding site on the capsid, providing insights about structural determinants of receptor and host specificity and evolution of these viruses.

## Introduction

*Trichodysplasia spinulosa*-associated Polyomavirus (TSPyV) was discovered in 2010 in facial skin samples from a patient with the skin disease *trichodysplasia spinulosa* (TS) [1]. TS is characterized by the development of folliculocentric papules and keratin spines, predominantly localized to the face and less frequently on the trunk and limbs of immunocompromised individuals [1, 2–4, 5]. The pathogenic mechanism of TSPyV during the symptomatic infection includes uncontrolled hyperproliferation of inner root sheath (IRS) cells, but molecular determinants underlying TSPyV infection and disease remain largely unknown [2,6,7]. Electron microscopic studies have confirmed the presence of icosahedral viral particles in affected hair follicles [2,4,8,9]. However, high viral loads have been detected only in TS patients, whereas samples from the skin, plucked eyebrows, serum/plasma, and urine of immunocompetent and-compromised individuals were mostly negative for TSPyV DNA [1,6,10]. In contrast, seroprevalence values of about 70% within the human population suggest that initial infections with TSPyV occur during childhood [11–13], and thus persistent infections at undetectable levels or in undiscovered latent extracutaneous reservoirs are very likely [10,14]. The detection of TSPyV DNA in tonsillar samples from healthy individuals indicates that the virus infects lymphoid tissue establishing a persistent infection [10,14]. Viral shedding and spreading from this persistent site may then be crucial for transmission and reactivation during immunosuppression [14].

Among the recently discovered human members of the growing polyomavirus family, TSPyV and the carcinogenic Merkel Cell Polyomavirus (MCPyV) have gained particular attention due to clear links to a human disease or human cancer, respectively [15,16]. Furthermore, the skin-tropic Human Polyomavirus 7 (HPyV7) has recently been associated with thymic epithelial tumors [17]. While these three viruses share skin tropism, characteristics of the infection and pathogenicity seem to differ. For example, MCPyV is clonally integrated in the host cell genome in the majority of the neuroendocrine Merkel cell carcinomas (MCC) [15], but there is no evidence for genomic integration of TSPyV to date.

TSPyV has a 5232-nucleotide dsDNA genome encapsulated in its non-enveloped icosahedral capsid made up of the proteins VP1, VP2 and VP3 [1]. X-ray crystallographic studies of the pentameric major capsid proteins (VP1s) from several polyomaviruses have revealed a conserved jell-roll fold topology [18–27]. On the outer surface of the virion, structurally distinct loops connect the β-sheet core, and these loops are chiefly responsible for viral antigenicity. They also form a virus-host interaction platform that contributes to host range, cell tropism, viral spread, and pathogenicity. Although sialylated glycans are functional receptors for several polyomaviruses, the role and importance of these glycans for infectious entry remain unknown for other more recently discovered family members. The engagement of non-sialylated receptor types has been suggested in several cases [22,27].

Sialic acids are commonly connected by either α2,3- or α2,6-linkages to a galactose (Gal), by an α2,6-linkage to N-acetylgalactosamine (GalNAc) or via α2,8-linkages to one another. They are abundantly expressed on N- or O-linked glycoproteins as well as on gangliosides, and several chemical modifications are known [28]. The predominant sialic acid species in humans is α-5-N-acetylneuraminic acid (Neu5Ac), which is a central building block of cell surface receptors for many human viruses [29,30]. In contrast, α-5-N-glycolylneuraminic acid (Neu5Gc), the predominant type of sialic acid in many other mammals, cannot be synthesized by humans due to a species-specific inactivating deletion in the gene encoding the hydroxylase that converts CMP-Neu5Ac to CMP-Neu5Gc [31,32]. However, Neu5Gc can be metabolically incorporated into human tissues from dietary sources [33] and its role in receptor engagement by viruses and in defining viral tropism is only beginning to emerge [34].

The same binding region at the surface of VP1 is employed for the interaction with terminal sialic acids in all structurally investigated sialic acid-engaging polyomaviruses so far, but amino acid differences in or near the core binding pocket can modulate the recognition of specific sialylated glycan receptors or receptor motifs [30]. Subtle VP1 amino acid changes in the binding pocket can have a critical impact on infection and viral pathogenicity [35,36,24,26]. This is illustrated by a single amino residue mutation in the binding pocket of the human BK Polyomavirus (BKPyV), which allows for a switch in the ganglioside receptor specificity [24]. Affinity is also critical, as the closely related JC Polyomavirus (JCPyV) binds several ganglioside motifs, including GM1 and GD1b, but the increased affinity for the α2,6-linked lactoseries tetrasaccharide c (LSTc) is crucial for its function as a receptor [21,37]. Murine Polyomavirus (MPyV), MCPyV, B-lymphotropic Polyomavirus (LPyV), and Human Polyomavirus 9 (HPyV9) recognize sialic acids in distinct orientations within the conserved location of the binding pocket on VP1 by applying different interaction strategies [19,23,25,26]. In contrast, Human Polyomaviruses 6 (HPyV6) and 7 possess elongated VP1 surface loops that obstruct the Neu5Ac-binding region, indicating that these viruses utilize non-sialylated receptors [27].

To enhance our understanding of the cell and host tropism of TSPyV, and ultimately its pathogenicity, we aimed to shed light on molecular determinants and principles during the early steps of infection. Following cell attachment studies, we determined high-resolution X-ray structures of TSPyV VP1 pentamers alone and in complex with glycans bearing α2,3- or α2,6-linked terminal Neu5Ac. TSPyV engages sialic acid receptors but the virus uses a novel binding site on VP1 that is shifted in comparison to all other structurally characterized polyomaviruses so far. Cell-based studies demonstrate the relevance of this binding site for cell attachment and infectivity, and also suggest that glycolipids rather than N- and O-linked glycoproteins play an important role during TSPyV infection. In conclusion, our findings highlight the complexity and plasticity of virus-glycan receptor interactions, and provide clues about the determinants of host specificity and evolution of TSPyV.

## Results

### Sialic acids promote TSPyV VP1 cell attachment

**Fig 1:**
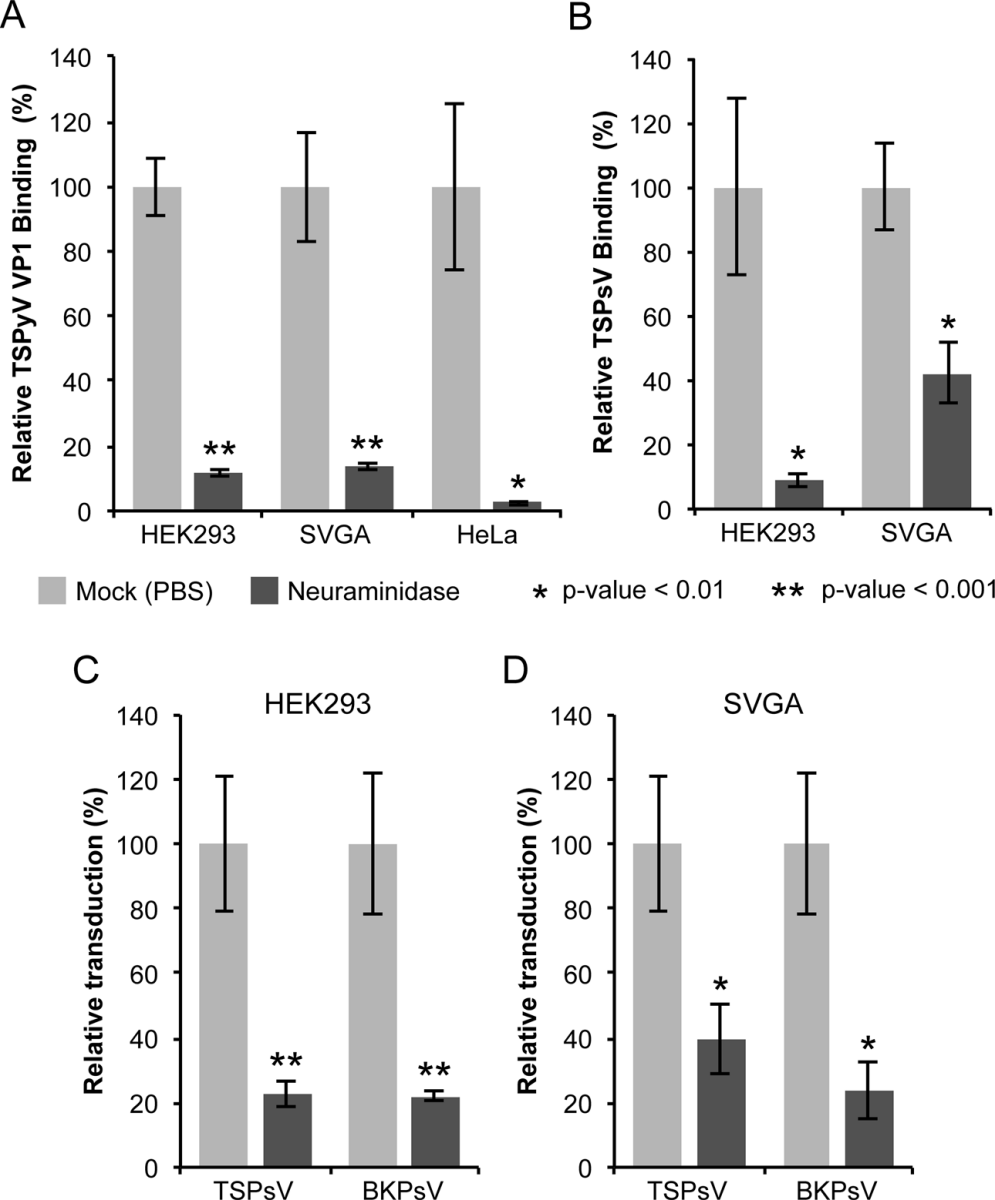
Cell surface glycans featuring terminal sialic acids promote TSPyV attachment and pseudovirus infection. (A) Binding of Alexa Fluor 488-conjugated TSPyV VP1 pentamers to mock (PBS) or with 1 U/ml *Clostridium perfringens* type V neuraminidase pre-treated cells was analysed by flow cytometry. 30,000 gated events were measured for each sample. The average from three independent experiments is shown, and error bars indicate the standard deviation. Data was standardized to signals of mock treated cells alone. S1 Fig shows non-standardized data of individual experimental replicates. (B) Attachment of Alexa Fluor 488-conjugated TSPsV to mock (PBS) or with 1 U/ml *Clostridium perfringens* type V neuraminidase pre-treated HEK293 and SVGA cells. The analysis was carried out by flow cytometry with 10,000 events measured for each sample. The average from three replicates is shown, and error bars indicate the standard deviation. (C-D) TSPyV pseudovirus (TSPsV) infections of HEK293 and SVGA cells (mock-treated cells and cells incubated with *Clostridium perfringens* neuraminidase for 30 min prior to PsV infection). BK Polyomavirus pseudovirus (BKPsV) was used as positive control for a neuraminidase-sensitive infection. The efficiency of the PsV infection was measured by transduction of the reporter plasmid phGluc coding for a secreted form of *Gaussia* luciferase 72 h post infection by detection of the secreted luciferase (measured in relative light units, RLUs using the BioLux *Gaussia* Luciferase Assay Kit). PsV experiments were done in quadruplicate and repeated three times and the average relative transduction is shown. Error bars indicate standard deviations and statistic analysis was performed using the two-tailed unpaired t test. The data was standardized to mock PsV transfections. The mock PsV control was generated by harvesting HEK293TT cells transfected with control plasmid instead of the capsid expression plasmids and then purifying according to the PsV purification protocol to measure background signal and non-specific transfer of luciferase to infected cells.

To determine if TSPyV interacts with sialic acids on cell surfaces, we first examined binding of unassembled TSPyV VP1 pentamers to cultured human cells by flow cytometry (Fig 1A and S1 Fig). For these experiments, TSPyV VP1 was recombinantly expressed in *E*. *coli*. The VP1 protein forms the characteristic homopentamer but cannot assemble further into viral capsids due to N- and C-terminal truncations of the expression construct. Cells were mock treated or incubated with *Clostridium perfringens* neuraminidase type V to remove terminal α2,3-, α2,6-, and α2,8-linked sialic acids from the cell surface prior to the flow cytometry experiment. The binding signal of TSPyV VP1 pentamers to enzyme-treated cells is significantly reduced compared to binding to mock-treated cells (Fig 1A and S1 Fig). BKPyV VP1 pentamers were used as positive control for neuraminidase-sensitive cell attachment (S1 Fig).

### Sialic acids are important for TSPyV pseudovirus infection

Next, we investigated the role of sialic acids in TSPyV infection. To our knowledge, a cell culture system for growing TSPyV is not available. However, virus-like particles (VLPs) or reporter vector particles, known also as pseudoviruses (PsV), have been generated for other polyomaviruses and are useful model systems for infection studies reflecting the viral tropisms [38–40,36]. We therefore developed a TSPyV pseudovirus system (TSPsV), which uses the TSPyV structural proteins expressed in the producer cells HEK293TT to package a reporter plasmid coding for secreted luciferase that can be detected by relative luminescence units (RLU) for quantification of PsV infection [39,40]. A mock control for PsV infections was generated by transforming the reporter plasmid together with a control plasmid instead of plasmids coding for structural proteins into producer cells. First, multiple common cell lines were screened for efficient transduction of the reporter plasmid to identify suitable cell lines for TSPsV infection studies (S2 Fig). Significant transduction in comparison to the mock transfection control was seen for HEK293 cells and the human glial cell line SVGA, but not for HeLa, monkey kidney (Vero) and Chinese Hamster Ovary (CHO) cells. TSPsV cell attachment and transduction in SVGA and HEK293 is sensitive to pretreatment with neuraminidase (Fig 1B–1D). Together with the cell binding analysis, the PsV infection studies suggest that TSPyV engages sialic acids on the cell surface during viral attachment and prior to infectious entry.

### Crystal structure of TSPyV VP1 pentamers in complex with α2,3- and α2,6-linked sialylated glycan motifs

**Fig 2:**
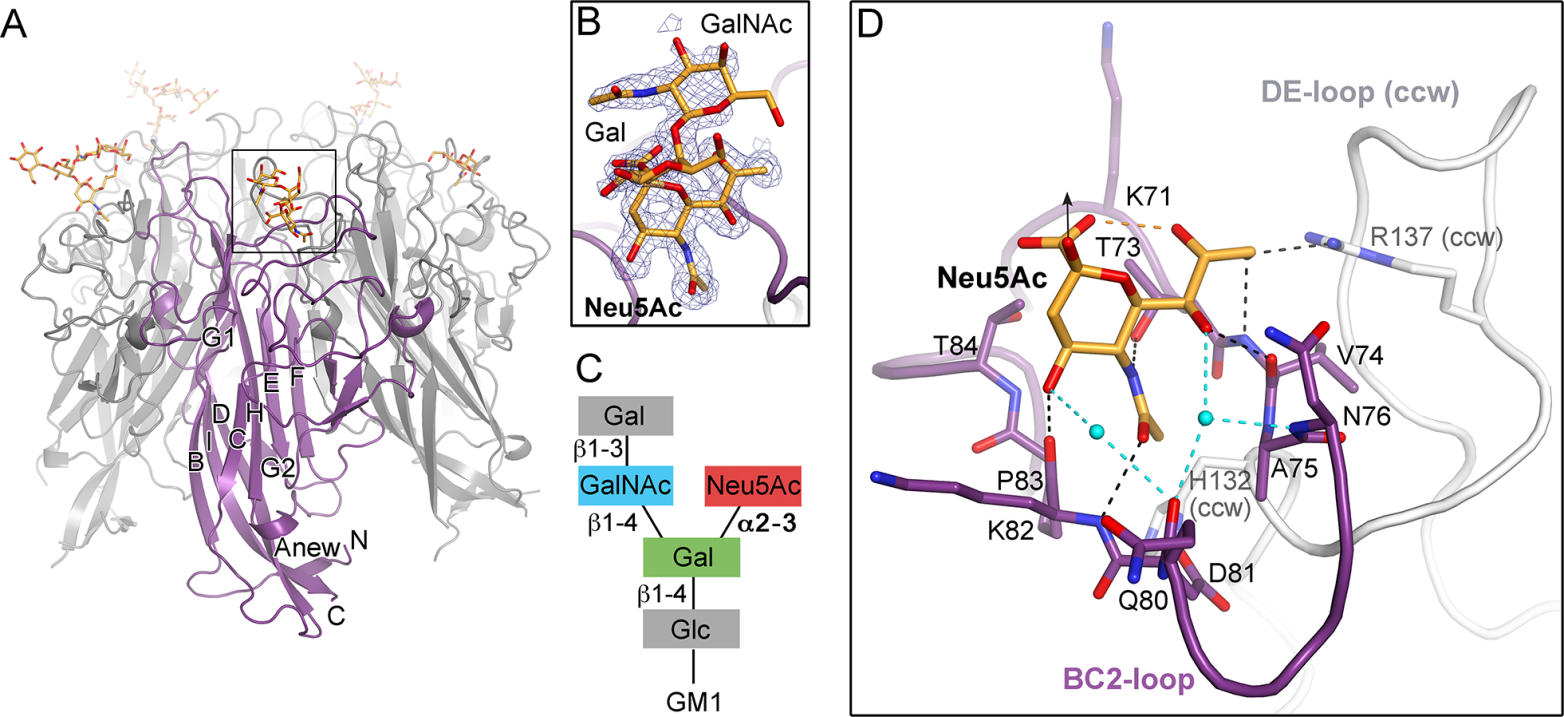
TSPyV VP1 specifically binds to terminal Neu5Ac. (A) Crystal structure of the unassembled TSPyV VP1 pentamer in complex with the GM1 glycan. The TSPyV VP1 pentamer is shown in cartoon representation with one monomer depicted in purple. Glycan residues are shown in stick representations and glycan atoms are coloured according to the atom type, with oxygen in red, nitrogen in blue, and carbon in orange. (B) Close-up view of the Neu5Ac binding site. The simulated annealing omit difference map contoured at 3.0 σ is shown with a radius of 2.0 Å around glycan residues Neu5Ac-α2,3-Gal-β1,4GalNAc of the GM1 glycan. (C) Schematic representation of the GM1 glycan. Glycan rings built into electron density in panel B are highlighted in colours. (D) Interaction between TSPyV VP1 and the terminal Neu5Ac residue of the GM1 glycan in a binding site that is formed by the BC2-loop. Side chains and backbone atoms of VP1 interacting with the glycan are shown in stick representation, and atoms are coloured as in panel A. Water molecules are shown as spheres, hydrogen bonds between VP1 and Neu5Ac are drawn as black dashed lines, water-mediated contacts between VP1 and Neu5Ac are depicted in cyan, and intramolecular Neu5Ac hydrogen bonds are shown in orange.

To establish a platform for understanding the specificity of the interaction with sialic acid, we solved crystal structures of TSPyV VP1 alone and in complex with three different sialylated compounds, the branched α2,3-linked GM1 pentasaccharide and the linear glycans α2,3- and α2,6-sialyllactose (3’SL and 6’SL, respectively) (Table 1 and Fig 2). TSPyV VP1 pentamers were co-crystallized in the presence of either 10 mM GM1 glycan or 10 mM 6’SL, while preformed native TSPyV VP1 pentamer crystals were derivatized by soaking them in a 10 mM 3’SL solution for complex formation. As is typical for polyomaviruses [18–27], the TSPyV VP1 monomer adopts the iconic jelly-roll fold with a conserved core comprising two β-sheets that are formed by strands B, I, D, G and C, H, E, F, respectively (Fig 2A). Extensive loops connect the strands on the top of the VP1 pentamer and are named BC-, DE-, HI- and EF-loops according to the strands they connect. The long BC-loop folds into two different directions on top of the protein and is divided for clarity into BC1-loop, BC-linker and BC2-loop [18,22]. In the unbiased difference (F_obs_-F_calc_) electron density map of the glycan complex structures, we observed well-defined electron density for the terminal Neu5Ac residues of 3’SL, 6’SL and the GM1 glycan. The highest occupancy was obtained for GM1 in all five binding sites of the VP1 pentamer in the asymmetric unit, and so additional glycan residues besides Neu5Ac could be built unambiguously into well-defined electron density (Fig 2B). Nevertheless, three of the five binding sites were not considered for further structural analysis because the ligand participates in crystal contacts. In the remaining two sites of the VP1 pentamer, the Neu5Ac residue and the GalNAcβ1-3(Neu5Acα2–3)Gal trisaccharide (Fig 2B and 2C), respectively, are well-defined by electron density and distant from crystal contacts. These two binding sites are therefore discussed below in detail.

**Table 1:** Crystallographic data collection and structure refinement statistics.

	TSPyV VP1	TSPyV VP1—GM1 glycan	TSPyV VP1–6’SL	TSPyV VP1–3’SL
PDB code	4U5Z	4U60	4U61	4U62
**Data collection**				
Space group	P2_1_	P2_1_2_1_2	P2_1_2_1_2_1_	P2_1_
Unit cell				
a, b, c [Å]	64.10, 153.64, 143.98	144.99, 152.05, 67.97	139.56, 146.35, 151.54	66.24, 152.84, 147.24
β [°]	91.83	90.00	90.00	92.34
Resolution [Å]	30–2.10 (2.16–2.10)	50–1.50 (1.54–1.50)	30–1.65 (1.69–1.65)	40–1.55 (1.59–1.55)
Unique reflections	158,871 (11,115)	238,315 (17,410)	368,928 (27,122)	412,698 (29,151)
Total reflections	749,166 (48,901)	1,312,931 (92,629)	3,423,638 (224,558)	1,584,158 (106,332)
R_meas_ [%]	9.0 (94.9)	8.1 (88.2)	9.5 (98.6)	6.2 (70.8)
I/σI	12.0 (1.7)	16.0 (2.0)	15.8 (2.3)	13.4 (2.2)
CC_1/2_ [%]	99.8 (65.2)	99.9 (66.3)	99.9 (75.3)	99.9 (76.3)
Completeness [%]	98.3 (92.8)	99.4 (99.0)	99.7 (100.0)	97.8 (93.5)
Wilson B factors [Å^2^]	44.1	21.5	27.3	29.2
**Refinement**				
R_work_/R_free_ [%]	20.5/24.9	15.8/18.0	16.5/18.5	15.5/18.0
No. of atoms				
Protein	20,375	10,611	20,747	20,892
Glycans	-	251	146	63
Water	551	1,637	2,267	2,355
Other solvent molecules*	-	74	24	46
Average B factors [Å^2^]				
Protein	43.0	17.3	23.5	27.5
Glycans	-	24.2	35.5	38.4
Water	38.5	26.9	30.6	32.4
Other solvent molecules*	-	33.2	34.0	37.0
RMSD				
Bond length [Å]	0.008	0.009	0.008	0.009
Bond angles [°]	1.273	1.419	1.309	1.356
Ramachandran plot				
Favored [%]	96.0	95.7	97.0	97.1
Allowed [%]	4.0	4.3	3.0	2.9

Values for the highest resolution shell are given in parentheses. R_free_ was calculated with 5% of the data. RMSD, root-mean-square deviation. The Ramachandran plot statistics were calculated with MolProbity [69].

*Other solvent molecules are glycerol and 1,2-ethandiol.

### Specific recognition of terminal sialic acids

The N-acetyl group of Neu5Ac inserts into a shallow cavity that is built by the BC2-loop on top of a VP1 monomer, thereby forming hydrogen bonds via its NH-group towards the hydroxyl side chain of threonine 73 and via its oxygen with the backbone amino group of lysine 82 (Fig 2D). The side chain of the arginine residue 137 from the DE-loop of the counterclockwise (ccw) VP1 monomer interacts with the glycerol chain of Neu5Ac. The backbone amino and carbonyl groups of residue V74 recognize the glycerol chain at one side, and the carbonyl group of residue K82 contacts the O4 of Neu5Ac on the other side of the BC2-loop. Residue H132 of the ccw monomer forms the bottom of the shallow binding groove so that the methyl group of Neu5Ac is recognized via hydrophobic interactions by the non-polar part of the ccw H132 side and by BC2-loop residues A75 and P83. Additionally, water-mediated hydrogen bonds are found between O4 and the glycerol chain of Neu5Ac and the VP1 backbone.

The VP1 surface area that is buried by the Neu5Ac interaction comprises 197 Å^2^ [41]. Because of the inherent flexibility of glycosidic linkages, complex glycans do not have a single, well-defined three-dimensional structure in solution and the mobility of the sugar rings are reflected in their temperature factors (B-factors). The average B-factor of the Neu5Ac ring of GM1 is in the same range as those of the neighboring protein residues, indicating that the occupancy is near 1.0 for the ligand. In contrast, the GalNAc and the branched Gal ring have elevated B-factors. This is in agreement with their lack of contacts to protein residues and resultant higher mobility. Neu5Ac moieties of 3’SL, 6’SL, and the branched GM1 glycan are bound in an identical manner in all three glycan-VP1 complex structures. Interactions with glycan residues other than the terminal Neu5Ac were not observed in any of the three complex structures. However, the branched “ganglioside core” GalNAcβ1-3(Neu5Acα2–3)Gal is likely more restricted in its conformational mobility than the linear glycan ligands 3’SL and 6’SL, and this could explain the better defined density for the GM1 glycan.

We next performed saturation transfer difference (STD) NMR spectroscopy experiments to provide evidence for the observed interaction between VP1 and the GM1 glycan in solution (S3 Fig). In this approach, saturation from a macromolecule, such as the VP1 pentamer, is transferred to a bound small-molecule ligand, such as a glycan, within a distance of up to 5 Å from the protein. Thus, this technique can be used to identify the protein-bound parts of the ligand [42]. We found that TSPyV VP1 interacts with the GM1 glycan in solution. Protons of the engaged glycerol chain (H7 and H9) and the methyl group (Me) of the N-acetyl chain of Neu5Ac are readily identified in the STD NMR difference spectrum. These findings are in good agreement with the crystal structure analysis, where mainly interactions with the terminal Neu5Ac and in particular with its glycerol and acetyl groups were observed. Protons of the α2,3-linked Gal and the GalNAc residues receive comparably little magnetization, and these protons are also not engaged by VP1 in the crystal structure.

### The sialic acid binding site on TSPyV VP1 mediates cell attachment and infection

In order to verify the functionality of the binding site on TSPyV VP1 for sialic acid-dependent cell binding, mutations in the sialic acid binding site were introduced into the unassembled TSPyV VP1 pentamer construct. To analyze the importance of threonine 73, which recognizes the N-acetyl group of Neu5Ac, this residue was mutated to alanine (T73A) in order to remove the hydrogen bond, and to glutamine (T73Q) and glutamic acid (T73E) in order to introduce steric hindrance. In addition, residues 71, 84, and 137 were also mutated (K71L, T84A, and R137A). The design of relevant VP1 mutations was limited because the TSPyV VP1-Neu5Ac interactions are mainly mediated by the protein backbone.

**Fig 3:**
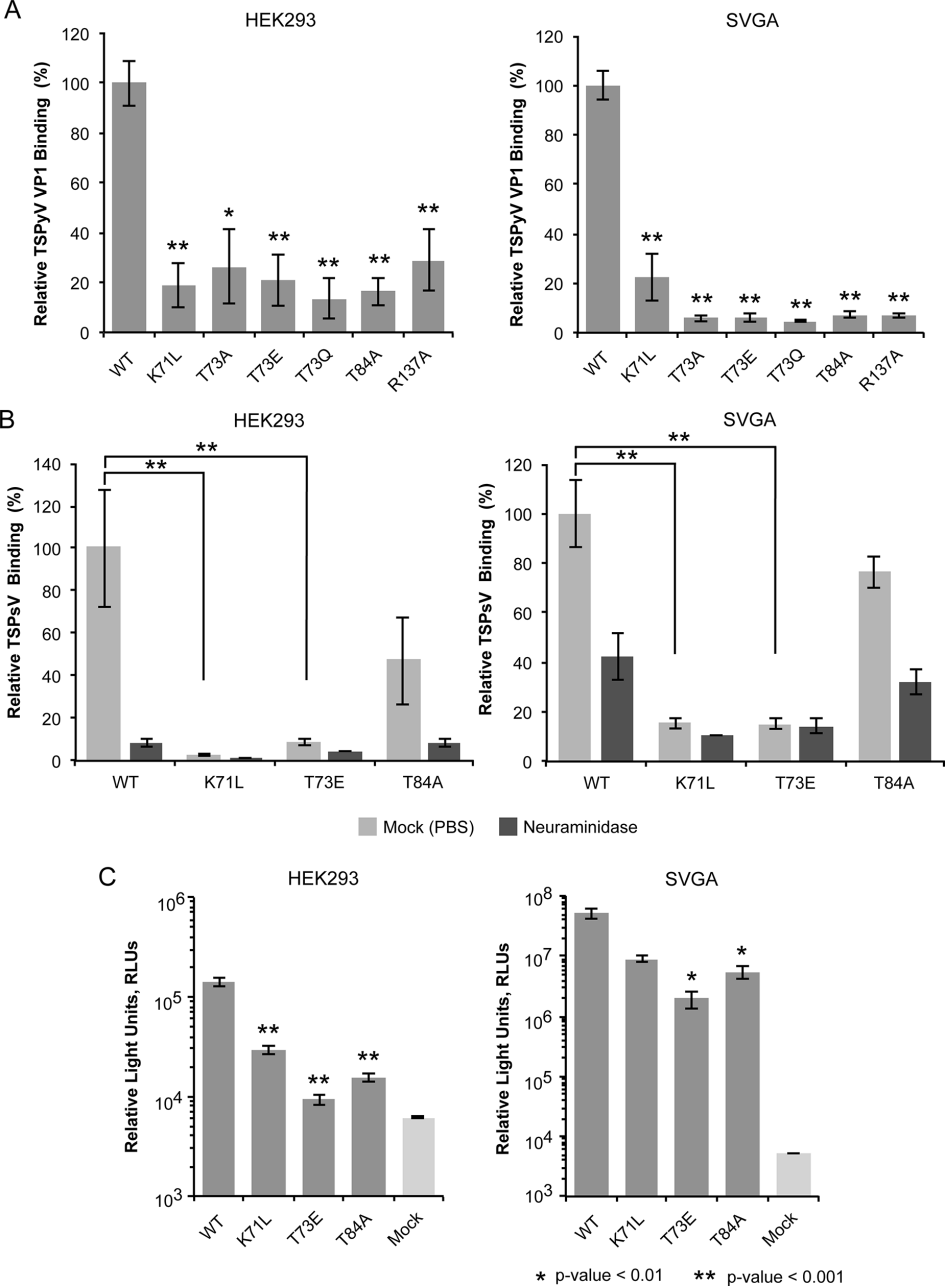
Cell binding and PsV infection of TSPyV mutants. (A) Cell binding analysis of TSPyV wild type (WT) and mutant VP1 pentamers to HEK293 and SVGA cells by flow cytometry. VP1 pentamers were covalently thio-labelled using Alexa Fluor 488 C5 maleimide. The histogram depicts the relative fluorescence signal of mutants compared to the binding signal of wild type pentamers. Data was measured in three independent experiments and was standardized for the background signal from cells alone. Error bars indicate standard deviations. (B) WT and mutant TSPsV binding (Alexa Fluor 488 amine-conjugated) to HEK293 and SVGA cells (mock-treated cells and cells incubated with *Clostridium perfringens* neuraminidase for 30 min prior to PsV infection) examined by flow cytometry. Relative fluorescence signals of mutants compared to the binding signal of WT PsV were plotted and error bars indicate standard deviations for three experimental replicates. Labeled PsV content was normalized by western blotting detection with purified PAB597, a hybridoma supernatant that produces a monoclonal antibody against JCPyV VP1, which cross-reacts with TSPyV VP1. The two-tailed unpaired t test was performed for binding of WT and mutant TSPsVs to mock-treated cells. (C) TSPsV infection of HEK293 and SVGA cells assayed 72 h post infection by detection of the secreted luciferase due to transduction of the reporter plasmid phGluc. The luciferase was quantified using a BioLux *Gaussia* Luciferase Assay Kit. The secreted luciferase catalyzes a photo-oxidation that emits light, which is measured in relative light units, RLUs. RLUs are given in logarithmic scale. Mock PsV infections were done with a control sample obtained according the PsV purification protocol from cells only transfected with phGluc and control plasmid measure background and nonspecific transfer of luciferase. PsV experiments were done in quadruplicate and repeated two times. Statistic analysis was performed using the two-tailed unpaired t test.

We used a thio-labeling strategy to standardize the labeling efficiency of TSPyV VP1 wild type and mutants for the flow cytometry experiment. TSPyV possesses a conserved cysteine, C107, within the CD-loop of VP1. This cysteine lies at the base of the pentamer, distant from the ligand binding site, and while it is the only solvent-exposed cysteine in the pentamer available for thio-labeling, it faces towards the interior and is not exposed in the assembled virus. For all mutants, the binding to HEK293 and SVG-A was significantly reduced (Fig 3A). Next, three of the mutations (K71L, T73E, and T84A) were introduced into TSPsV particles. All three mutations reduce cell binding of PsV and infectivity in HEK293 and SVGA cells (Fig 3B and 3C). The PsV infection assay reveals small differences between the mutants with T73E having the largest influence on infectivity in both cell lines tested. This finding is in accord with the VP1-Neu5Ac interaction seen in our structures. Residue T73 recognizes the N-acetyl group of Neu5Ac and has to be considered a central component of the binding site. Although none of the selected mutations abolishes PsV infection completely, each one of them reduces binding of VP1 pentamers and PsVs, indicating that the identified binding site on TSPyV clearly mediates attachment to sialylated glycans on cells, and thus defines an important event during early steps of TSPyV infection.

### A new location of the sialic binding site on the polyomavirus VP1 protein

**Fig 4:**
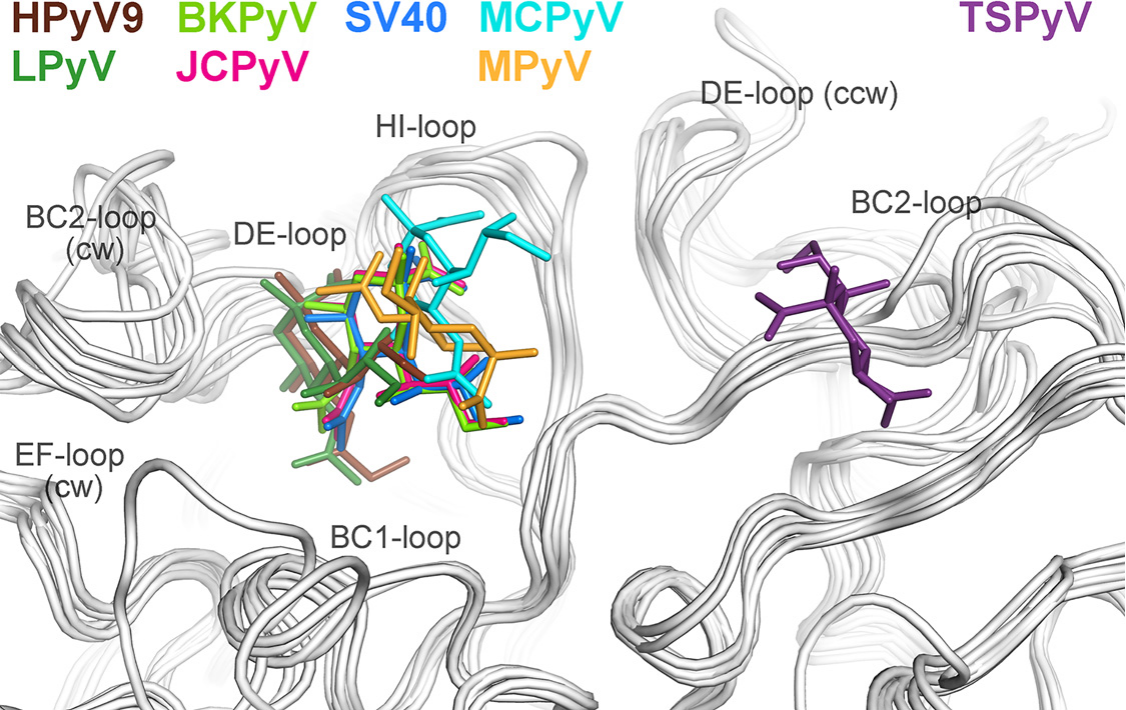
Unique location of the TSPyV Neu5Ac binding site. The location of the Neu5Ac binding site on TSPyV VP1 is shifted compared to binding sites of polyomaviruses SV40, BKPyV, JCPyV, MCPy, LPyV, HPyV9 and MPyV, which all engage sialylated glycans by employing a Neu5Ac binding site in a conserved location on VP1. For clarity, only terminal Neu5Ac residues are shown in stick representations and are coloured according as assigned for each virus. TSPyV VP1-GM1 glycan (purple), SV40 VP1-GM1 glycan (blue; pdb 3BWR), BKPyV VP1-GD3 glycan (light green; pdb 4MJ0), JCPyV VP1-LSTc pentasaccharide (pink; pdb 3NXD), MCPyV VP1-GD1a glycan (cyan; pdb 4FMJ), LPyV-α2,3-sialyllactose (dark green; pdb 4MBY), HPyV9 VP1-α2,3-Neu5Gc-sialyllactose (brown; pdb 4POT), MPyV VP1-Neu5Ac-α2,3-Gal-β1,3-[α2,6-Neu5Ac]–GlcNAc-β1,3-Gal-β1,4-Glc (orange; pdb 1VPS).

To date, X-ray structures of seven different polyomavirus VP1 proteins in complex with sialylated glycans have been solved [19–21,23–26]. The human JCPyV, BKPyV, MCPyV and HPyV9 viruses, but also the two simian viruses LPyV and SV40 and the murine virus MPyV engage sialic acids of their specific glycan receptor motifs in the same general area of VP1 (Fig 4). This shallow binding site is located at the interface between two monomers, with contributions from the DE-, HI- and BC1-loops and the BC-linker of one monomer and ccw BC2- and EF-loops from the neighboring VP1 chain. In contrast, TSPyV engages Neu5Ac in a unique, exposed binding site that lies about 18 Å away from the sialic-acid binding groove of all other polyomaviruses. This new binding site is formed by the BC2-loop of a single VP1 monomer on top of the pentamer. The comparison of all seven structures reveals that TSPyV VP1 does not differ drastically in its amino acid sequence, length and fold of the surface loops. Full-length TSPyV VP1 shares 52–61% amino sequence identity with these seven other VP1 proteins. BKPyV and JCPyV VP1 are most closely related to TSPyV VP1 in terms of the overall VP1 structures as well as the amino acid sequences with 52 and 54% VP1 sequence identities, respectively. The TSPyV VP1 structure superposes on the BKPyV and JCPyV VP1 structures with small root-mean-square deviation (RMSD) values of about 0.8 Å (calculated for Cα atoms of the VP1 monomers).

### Structural basis for the relocation of the sialic binding site on VP1

**Fig 5:**
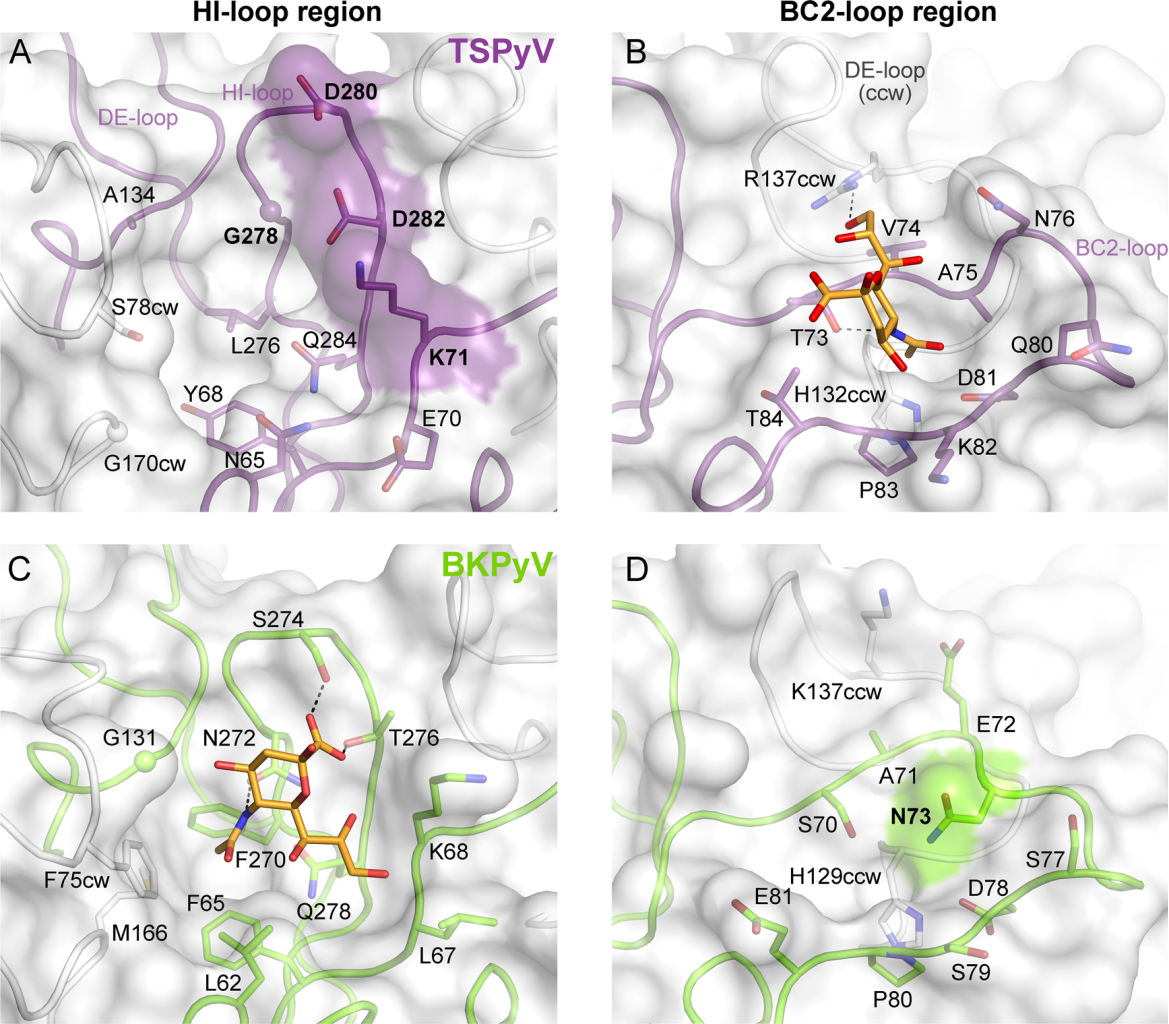
Comparison of the Neu5Ac binding sites of HI- loop and BC2- loop regions. (A, C) HI-loop regions of TSPyV VP1 and of BKPyV VP1 in complex with the GD3 glycan (pdb 4MJ0), are shown in the same orientation. The terminal Neu5Ac of GD3 is show in orange sticks. Residues that do not allow interaction with Neu5Ac in a manner similar to BKPyV are colored on the surface of VP1. Direct hydrogen bonds between BKPyV VP1 side chain residues and Neu5Ac are depicted as dashed lines. (B, D) BC2-loop regions of TSPyV VP1 and BKPyV VP1 are shown. Direct hydrogen bonds between side chain residues of TSPyV VP1 and the terminal Neu5Ac of the GM1 glycan and an intramolecular BKPyV VP1 hydrogen bond are shown as black dashed lines. The structures were aligned using their Cα atoms. One VP1 monomer is highlighted in purple for TSPyV and light green for BKPyV, respectively.

To understand the molecular basis for the relocation of the Neu5Ac binding site we compared the TSPyV VP1-GM1 complex structure with known VP1-glycan complex structures in more detail. Usually, the terminal sialic acids is recognized by HI-loop residues of VP1 via direct interactions with the protruding functional groups of the Neu5Ac, the carboxylate group, the N-acetyl group and the glycerol chain [30]. In TSPyV VP1, only small differences in structure and amino acid sequence within the HI-loop binding region are sufficient to switch the location of the Neu5Ac binding site to the BC2-loop region as shown in an exemplary manner for the comparison with the BKPyV VP1-GD3 glycan structure (Fig 5). The groove forming the binding site for the terminal sialic acid in VP1 of BKPyV and other polyomaviruses next to the HI-loop is still present on TSPyV VP1. This groove is often hydrophobic at the bottom and recognizes the methyl group of N-acetyl chain of Neu5Ac [21,23–25] through a hydrophobic patch formed by residues L62, F65, F270 and F75cw of BKPyV VP1. With Y68 and L276, the equivalent region of TSPyV VP1 is similarly hydrophobic (Fig 5A and 5C). However, while the carboxylate group of Neu5Ac is recognized by two hydrogen bonds from side chains of residues S274 and T276 in BKPyV VP1, similar interactions are not possible in TSPyV as the recognition of the sialic acid carboxylate group appears to be electrostatically hindered through residues D280 and D282. In addition, residue G278 in TSPyV, which substitutes for residue N272 of BKPyV, cannot recognize the N-acetyl group of Neu5Ac in the HI-binding loop. Residue K71 may also interfere with binding of Neu5Ac in the HI-loop region of TSPyV VP1, although the K71 side chain has elevated temperature factors. On the other hand, the shifted Neu5Ac-binding site in TSPyV VP1 is unable to bind sialic acid in BKPyV VP1 as residue N73 forms a hydrogen bonds with residue S70, thereby widening the BC2-loop and interfering with Neu5Ac binding. Hence, subtle differences seem to modulate the recognition of Neu5Ac both in the HI-loop region and also in the BC2-loop region (Fig 5B and 5D).

### Glycolipids are important for TSPyV infection

**Fig 6:**
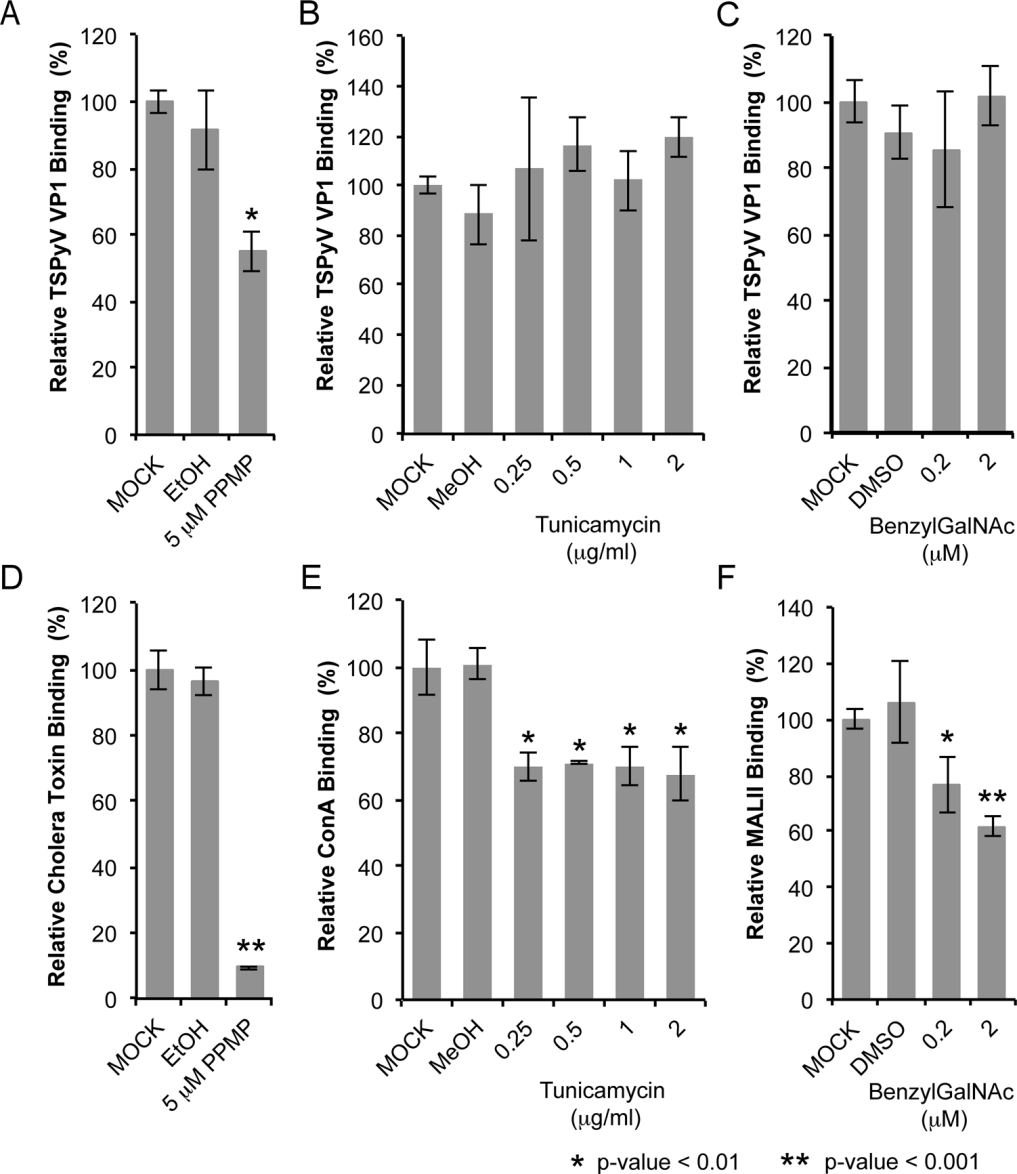
TSPyV binds to sialic acid containing glycolipids. Cell binding analysis of thio-labelled Alexa Fluor 488 TSPyV VP1 pentamers to HEK293 cells treated with inhibitors interfering with the synthesis of glycosphingolipids, N. and O-linked glycoproteins. Cells were treated with (A, D) PPMP, (B, E) tunicamycin, (C, F) BenzylGalNAc, and the respective carrier controls. The binding of TSPyV pentamers was standardized to binding signals to mock (PBS) treated cells. 30,000 gated events were measured for each sample. Cell binding of cholera toxin B subunit and the lectins ConA and MALII was used to monitor the effect of the inhibitors on the cellular expression of glycans. Data was measured in three independent experiments. Error bars indicate standard deviations.

Sialic acids are abundantly expressed on N- or O-linked glycoproteins, and they are also key components of glycolipids and in particular gangliosides. In order to elucidate the cellular scaffold of the sialic acids engaged by TSPyV we treated HEK293 cells with inhibitors interfering with the synthesis of these molecular scaffolds for terminal sialic acids and monitored TSPyV pentamers binding (Fig 6). Tunicamycin inhibits the synthesis of N-linked glycans, Benzyl-α-N-acetylgalactosamine (BenzylGalNAc) specifically blocks the synthesis of O-linked glycans, and D,L-threo-phenyl-2-hexadecanoylamino-3-morpholino-propanol (PPMP) is a structural analogue of a ceramide and inhibits the UDP-glucose-ceramide glucosyltransferase leading to decreased glycosphingolipid expression on cells. The effects of the cell treatment were assessed with two positive controls: (i) cholera toxin B subunit, which binds GM1 gangliosides, and (i) the lectins Concanavalin A (ConA) and *Maackia amurensis* II (MALII), which bind to N- and O-glycans. In addition, the toxicity of either drug was monitored using a cell proliferation assay, confirming that drug treatments did not reduce the cell viability. Tunicamycin and BenzylGalNAc did not affect cell binding of TSPyV pentamers, whereas a significant reduction of attachment to HEK293 cells was observed after treatment with PPMP. Similar flow cytometry experiments were carried out with SVGA cells. Tunicamycin and BenzylGalNAc treatments did not reduced TSPyV VP1 pentamer binding to SVGA cells. However, cholera toxin B subunit and TSPyV VP1 pentamer binding to PPMP-treated SVGA cells was also not significantly reduced, suggesting that inhibition of the glycosphingolipid expression by PPMP is not very effective in this cell line.

**Fig 7:**
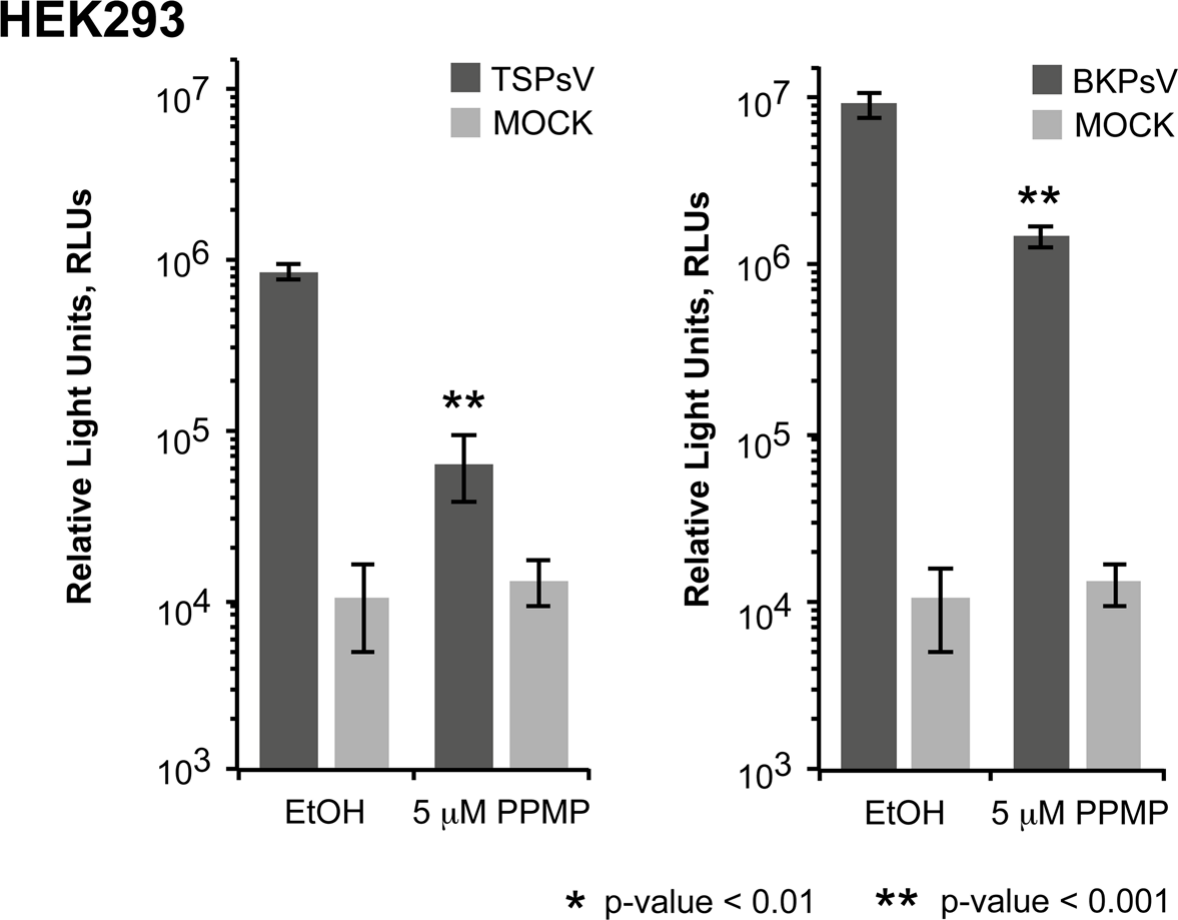
Glycolipids are important for TSPsV infection. TSPsV infection of HEK293 pre-treated with PPMP or the carrier control for 6 days was assayed 72 h post infection by quantification of the secreted luciferase due to transduction of the reporter plasmid phGluc. BKPsV were used as a positive control for a ganglioside-dependent infection. Mock PsV infections were done with a control sample obtained according the PsV protocol from cells only transfected with phGluc and control plasmid to test for background signal of the luciferase assay for uninfected cells. PsV experiments were done in quadruplicate and repeated three times. The results from one representative quadruplicate experiment is shown here as an example. Statistic analysis was performed using the two-tailed unpaired t test.

TSPsV transduction in PPMP-treated HEK293 cells was significantly impaired, suggesting that glycolipids are important for TSPyV infection of these cells (Fig 7). Additionally, the human lung cell line A549 could be infected with TSPsV resulting in a reduction after cell treatment with PPMP (S4 Fig). However, we could not observe reduced binding of TSPyV VP1 pentamer to A549 cells after PPMP treatment. Hence, these experiments establish that a glycolipid is likely important for viral entry, but TSPyV might also bind to other sialylated glycans on the cell surface during initial cell attachment, possibly in a cell type-specific manner.

### Phylogenetic and structural analysis highlights the close relationship to Orangutan Polyomavirus

**Fig 8:**
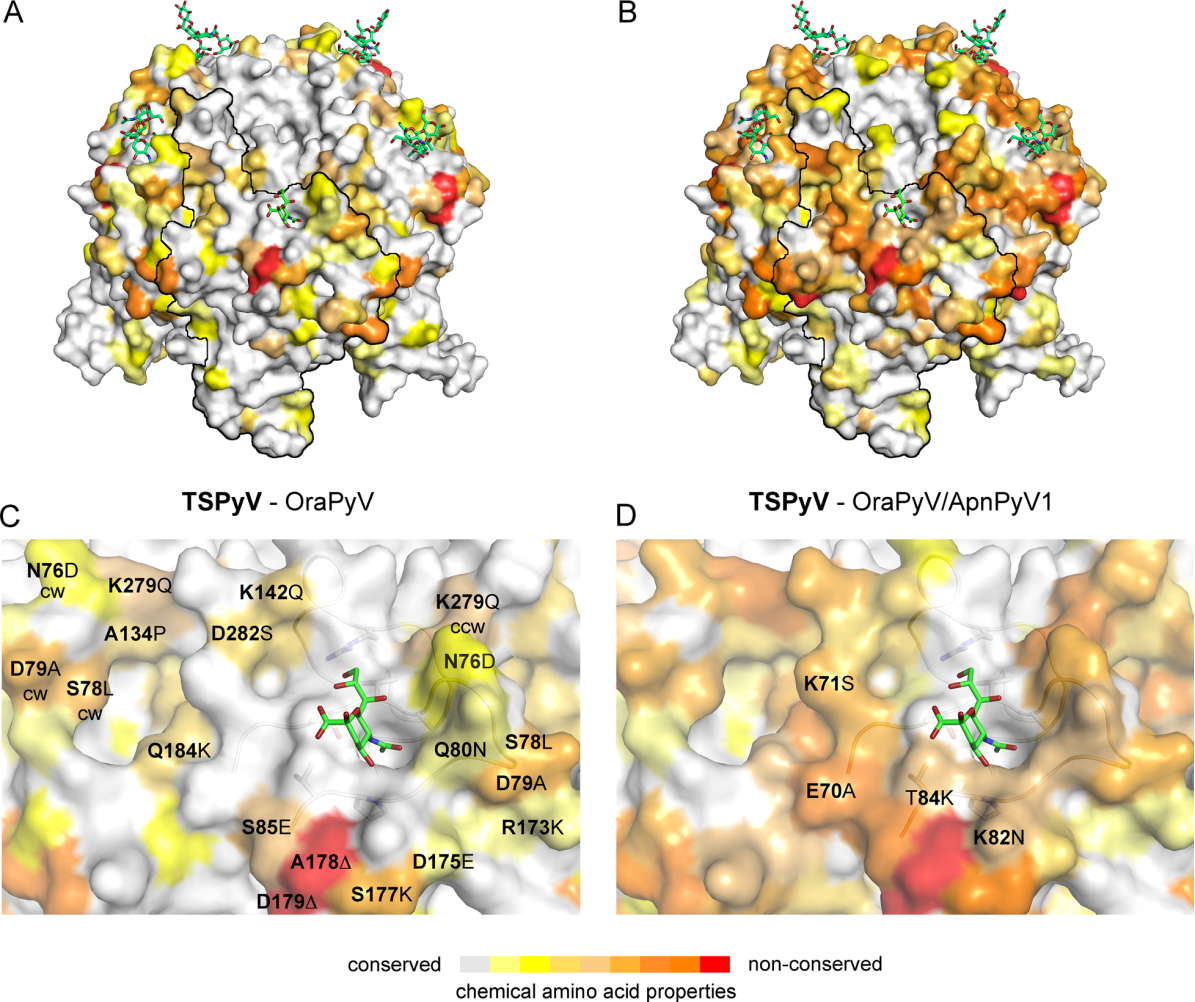
Conserved binding site for terminal Neu5Ac build up by the BC2-loop. Mapping amino acid differences between (A, C) TSPyV VP1 and OraPyV VP1 and (B, D) TSPyV, OraPyV and ApanPyV based on the TSPyV VP1-GM1 complex structure. Amino acid differences between the two or three closely related viruses are coloured according their conservations on the surface of TSPyV VP1. An amino acid sequence alignment was done with Clustal Omega. JalView [67] was also used to assign values for the respective conservation of the chemical acid amino acid properties from 10 (conserved; grey) to 0 (non-conserved; red). Values of 2 to 0 are colored in red. (C-D) Close-up views of the HI- and BC2-loop regions. The backbone of the BC2- and the ccw DE-loops are depicted in cartoon representations. Side chains of conserved key amino acids within the BC2-binding site are shown in sticks in panel C.

The phylogenetic analysis based on VP1 amino acid sequence reveals that TSPyV is most closely related to two simian polyomaviruses, Bornean Orangutan Polyomavirus (OraPyV1) and Ateles paniscus polyomavirus 1 (ApanPyV1) [1,43]. TSPyV shares VP1 amino acid sequence identities of 79% and 69% with OraPyV and ApPyV1, respectively. To investigate common features and differences between capsids of these three viruses, we compared their VP1 sequences and mapped conservations onto the molecular surface of TSPyV VP1 (Fig 8). Differences occur predominantly on the top surface of the VP1 pentamer, which is especially accessible to antibodies in the context of the assembled virion. VP1 residues within the BC2-loop binding site are highly conserved in OraPyV (Fig 8A) but the HI-loop region differs in the human and in the monkey virus. The comparison of VP1 proteins from TSPyV, OraPyV and ApanPyV1 highlights that BC2-loop residues T73, V74 and P83 are conserved in contrast to the rest of the upper VP1 surface. In addition, DE-loop residue R137, which interacts with the glycerol chain of Neu5Ac in TSPyV VP1, is also present in OraPyV and ApanPyV1 VP1. Consequently, based on the structure-based sequence alignment, we predict that TSPyV shares its sialic acid binding site with OraPyV and ApanPyV1.

## Discussion

Although a large number of new polyomavirus family members have been identified in recent years [16], details about their receptor-binding specificities and cell attachment strategies are not known with the exception of MCPyV and HPyV9 [23,26]. We demonstrate here that the skin-tropic TSPyV binds sialylated glycans in a binding site formed by the BC2-loop on top of the VP1 protein that is not utilized by any other structurally characterized member of the polyomavirus family. This new site is highly exposed to solvent and, apart from its location, also unique in that the sialic acid carboxylate group is not engaged by the protein, in contrast to all other known virus-sialic acid interactions [44]. The binding site has functional relevance, as site-directed mutations decrease binding of VP1 pentamers and PsVs to cells as well as PsV transduction. However, since the introduced mutations did not abolish PsV transduction completely, we cannot rule out that additional glycan residues on sialylated oligosaccharides, a different type of glycans such as glycosaminoglycans, or proteinous receptors are involved in the initial steps of TSPyV infection.

Our structures show that TSPyV VP1 can interact with terminal sialic acids in α2,3- and α2,6-linkages. Due to the exposed location of the binding site, glycans bearing terminal α2,8-linked sialic acid may be engaged in a similar manner. It is likely that TSPyV recognizes a more complex glycan motif or receptor similar to other polyomavirus members on its target cells to mount an infection. This hypothesis is supported by the observation that TSPsV transduction of HEK293 and A549 cells is reduced after interfering with the glycolipid synthesis by drug treatment. However, the cell treatment with PPMP did only reduce binding of TSPyV VP1 pentamers to HEK293, but not to A549 cells suggesting that different glycans may support cell attachment in different cells lines. To identify the complete glycan receptor(s) for TSPyV, further studies are required such as glycan array screening or the use of cutaneous cell lines or primary cells for infection studies. Importantly, functional data are needed to define the role of glycolipids for the TSPyV infection of IRS cells resulting in hyperproliferation and TS in immunocompromised patients [7]. Compared to normal hair follicles, TS hair follicles seem devoid of normal hair shafts and papilla but include instead large numbers of eosinophilic, trichohyalin-positive IRS cells [45]. The composition of cell surface glycans in different skin cells, for example the expression of mostly non-sialylated glycoconjugates in hair follicles during the keratinization process, has been investigated by lectin histochemistry, but these studies are often incomplete [46–48]. In addition, quantitative data and detailed information about the prevalence of sialic acid-containing glycoconjugates and chemical modifications of sialic acids would advance an understanding of the tropism of TSPyV for IRS cells. With regard to sialic acids, keratinization has also been shown to include reduction of these glycan residues in the plasma membrane of feline hair cortex during the differentiation [49]. Thus, it remains to be investigated if additional factors besides sialic acids support TSPyV infection of these cells. However, it is also important to realize that TSPyV is able to infect other, non-cutaneous cell types, for example tonsillar tissue [14].

Previous functional and structural studies established that polyomaviruses have a plastic binding site for Neu5Ac in a highly conserved region on top of VP1 that can engage the Neu5Ac ring in at least four different orientations [30]. Exceedingly small alterations in or near this binding site of polyomavirus VP1 have been found to modulate receptor specificity, which can render cells refractory to infection [35,24] or are critical for pathogenicity [50,51,36]. The case of TSPyV provides an example of a different strategy for modulating receptor interactions, as the typical binding site is no longer accessible and a new, alternate site has been developed. This new site is likely also present in related polyomaviruses. Examples for virus families in which members use different sites to engage a similar glycan receptor epitope are rare and include reoviruses [52,53] and adenoviruses [54–56]. While the physiologic reason for such alternate binding sites is not clear in either of these cases, virus-glycan interactions seem well suited for such shifts as they are usually of low affinity and depend on only a few specific contacts that can easily be modulated.

Many polyomaviruses form ‘pairs’ in which a human and a non-human version are most similar in sequence and therefore most likely closely linked in evolution [43,57]. A recent example is the HPyV9-LPyV pair, which displays subtle differences in sialic acid specificity [25,26]. In most cases, however, we lack knowledge about how such pairs differ in structure and receptor recognition, and how differences might relate to host tropism [34]. The architecture and the location of the BC2-loop binding region in TSPyV is likely conserved in OraPyV and ApnPyV1, and it is tempting to speculate that sequence differences near the conserved HI-loop binding region modulate binding to more complex, and perhaps subtly differing, sialylated glycan structures expressed on cells in the respective hosts.

Our analysis of TSPyV glycan binding properties provides a useful platform for further studies to define role of glycans for the TSPyV entry pathway, cell and host tropism as well as pathogenicity in order to understand structural determinants of receptor and host switching events. Such studies will inform not only polyomavirus research but also help to provide guidelines for rationalizing the receptor-binding specificities and tropism of other glycan-binding viruses.

## Materials and Methods

### Cell culture

Cells were maintained in a humidified 37°C CO_2_ chamber in Dulbecco’s Modified Eagle Media (DMEM) or Minimum Essential Media (MEM) supplemented with 1% penicillin/streptomycin and 10% heat-inactivated fetal bovine serum (FBS). SVGA cells have been derived from the original SVG human glial cell line established by SV40 transformation of human fetal glial cells [58]. HEK293 cells (human embryonic kidney cell line), HeLa cells (human epithelial cell line derived from cervical carcinoma), Vero cells (monkey kidney epithelial cell line), CHO cells (epithelial cell line derived from hamster ovaries), and A549 cells (human lung epithelial) were purchased from the American Type Culture Collection (ATCC). HEK293TT cells are HEK cells transformed with two copies of SV40 large T antigen (a gift from Dan DiMaio, Yale University).

### Protein expression, purification and fluorescence labeling

cDNA sequence (GenBank accession code ADK12664) coding for amino acids 31–304 of TSPyV VP1 was amplified by PCR and cloned into the *Nhe*I and *Bam*HI restriction sites of pET28b (Novagen). To be consistent with the previously described polyomavirus VP1 pentamer structures and to facilitate structural comparisons, the amino acid numbering of TSPyV VP1 (amino acids 30–303) used here in this study excludes the N-terminal methionine. The N- and C-terminal truncated TSPyV VP1 expression construct possesses an N-terminal hexahistidine tag (His-tag) followed by a thrombin cleavage site. Site-directed mutagenesis was done using 5’ to 3’ primers as follows (mismatched nucleotides are highlighted in boldface):

K71L: CTAAGGACAACTATGGTTACAGTGAA**CT**AGTAACTGTTGCTAACAGCAG,

CTGCTGTTAGCAACAGTTACT**AG**TTCACTGTAACCATAGTTGTCCTTAG; T74A:

GGTTACAGTGAAAAAGTA**G**CTGTTGCTAACAGCAGTGACCAG, CTGGTCACTGCTGTTAGCAACAG**C**TACTTTTTCACTGTAACC;

T73E: GGTTACAGTGAAAAAGTA**GAA**GTTGCTAACAGCAGTGACCAGGACAAGC, GCTTGTCCTGGTCACTGCTGTTAGCAAC**TTC**TACTTTTTCACTGTAACC; T74Q: GGTTACAGTGAAAAAGTA**CAG**GTTGCTAACAGCAGTGACCAGGACAAGC, GCTTGTCCTGGTCACTGCTGTTAGCAAC**CTG**TACTTTTTCACTGTAACC T85A:

CAGTGACCAGGACAAGCCT**G**CTTCTGGAGAGATAC, GTATCTCTCCAGAAG**C**AGGCTTGTCCTGGTCACTG;

R137A: CTGGTAAATGTTCATATGGCTACTAAA**GC**AATGTATGATGACAAAGGTATTG,CAATACCTTTGTCATCATACATT**GC**TTTAGTAGCCATATGAACATTTACCAG

Expression was performed in *E*. *coli* BL21 (DE3), and His-tagged VP1 was purified by nickel affinity chromatography from the crude cell extract. For crystallization, the His-tag was cleaved off in solution using thrombin (GE Healthcare). Six non-native residues (GSHMAS) are present at the N-terminus of VP1 after thrombin cleavage. His-tagged and cleaved wild type and mutant VP1 pentamers were further purified by gel filtration (Superdex 200). Prior to flow cytometry experiments, His-tagged VP1 pentamers were labeled with Alexa Fluor 488 C5 maleimide (Invitrogen). VP1 pentamers (1 mg/ml) were incubated for 16–18 h with 80 mM dithiothreitol (DTT) at 4°C. Excess DTT was removed using 2 x 5 ml HiTrap Desalting columns (GE Healthcare) and then the dye (10 mM in 20 mM HEPES pH 7.0, 150 mM NaCl) was added dropwise by gently mixing to the protein solution (0.2–0.3 mg/ml in 20 mM HEPES pH 7.0, 150 mM NaCl) to give a 10 molar excess of the dye. The reaction was incubated for 18 h at 4°C. 10 mM DTT were added and excess of the dye and DTT were removed by desalting. The labeling efficiency was determined by absorption according to the manufacturer's protocol. The molecular ratio was about 1: 4 (VP1 pentamer: dye) for wild type and mutant VP1 pentamers.

### Flow cytometry experiments

Cells that were 80–90% confluent were detached non-enzymatically from flasks by incubation with Cellstripper (Corning) for 10–20 min at 37°C. Cells were washed twice with PBS. Next, 10^6^ cells were suspended in 75 μl of labeled wild type and mutant TSPyV VP1 pentamer solution (20 μg/ml in PBS) for incubation on ice for 2 h with agitation every 20 min. Cells were washed twice in PBS and then fixed in 500 μl of 1% paraformaldehyde. Analysis was done using a BD FACSCalibur (Becton, Dickinson and Company) flow cytometer equipped with a 488-nm excitation line. Between 30,000 to 50,000 gated events were measured for each sample. Data were analyzed using FlowJo software (Tree Star, Inc.).

The flow cytometry experiments with the PsV were performed on purified PsV labeled with Alexa Fluor 488 carboxylic acid succinimidyl ester (Invitrogen). After labeling, PsVs were dialyzed into 10 mM Tris pH 7.4, 50 mM NaCl, 0.01 mM CaCl_2_ using 10,000 MWCO Slide-A-Lyzer dialysis cassettes (Life Technologies) to remove excess dye.

Labeled PsV preparations were normalized for VP1 content by Western blot. 6 x 10^5^ cells were mixed with equal amounts of labeled PsV as determined by Western Blot (about 50 μg τοταλ προτειν, measured via absorption at 280 nm) in 50 μl allowed to bind on ice for 30 minutes, washed and resuspended in PBS and then analyzed as pentamers counting 10,000 events. Experiments were performed in triplicate.

Fluorescein-labeled Concanavalin A (ConA, Vector Laboratories, USA) was added at a concentration of 1 μg/ml in 100 μl PBS to 10^6^ cells and was incubated on ice for 1 h with agitation every 20 min. Biotin-labeled *Maackia amurensis* II lectin, (MALII; Vector Laboratories, USA) at a concentration of 1 μg/ml was incubated with 10^6^ cells in 100 μl PBS on ice for 1 h. In the case of MALII, cells were washed 2x in PBS and detection of MALII was carried out with Alexa Fluor 488-labeled Streptavidin (488-Strep, Invitrogen), which was added at 2 μg/ml to about 10^6^ cells in 100 μl PBS for 30 min. Mock treated cells were incubated with 488-Strep as a negative control. The cholera toxin subunit B Alexa Fluor 488 conjugate (Invitrogen) was added at 10–20 μg/ml in 100 μl PBS to 10^6^ cells and was incubated with 10^6^ cells in 100 μl PBS on ice for 2 h. Prior to measurement by flow cytometry, cells were washed again twice with PBS and were then fixed in 500 μl of PBS, 1% paraformaldehyde.

### PsV production

For expression in the human derived cell line HEK293TT, codon optimization of TSPyV VP1, VP2 and VP3 genes was performed according the guidelines from the National Cancer Institute Center for Cancer Research Lab of Cellular Oncology Technical Files (http://home.ccr.cancer.gov/LCO/production.asp). DNA was synthesized by BlueHeron Biotechnology, LLC (Bothell, WA, USA). The VP1 gene was subcloned into the pwP vector in place of the MPyV VP1 gene and VP2 and VP3 genes into the ph2p vectors in place of the respective MPyV genes. Sequences are based on VP1, VP2 and VP3 sequences (NCBI reference YP_003800006, YP_003800004, YP_003800005) from the full length TSPyV genome (NCBI reference NC_014361). The luciferase reporter vector phGluc expresses a secreted form of *Gaussia* luciferase under control of the EF1α promoter. The parent PsV plasmids were obtained from AddGene (Cambridge, MA, USA). Site-directed mutagenesis of PsV VP1 was done using 5’ to 3’ primers as follows (mismatched nucleotides are highlighted in boldface):

K71L: GATAATTACGGCTATTCCGAG**CT**GGTGACCGTCGCCAATTCATC; GATGAATTGGCGACGGTCACC**AG**CTCGGAATAGCCGTAATTATC;

T73E: CTATTCCGAGAAGGTG**GAA**GTCGCCAATTCATCCGATC; GATCGGATGAATTGGCGAC**TTC**CACCTTCTCGGAATAG;

T84A: CGATCAAGATAAACCC**G**CCAGCGGCGAAATCCCCAC; GTGGGGATTTCGCCGCTGG**C**GGGTTTATCTTGATCG;

TSPsV were purified by a sucrose gradient followed by a CsCl gradient as described earlier [59].

Titers of purified PsV were determined according the encapsidated reported plasmid phGluc for infections studies. PsV preparations were treated with DNAse I (New England Bioloabs) for 60 min. DNAse I was then inactivated by 75°C for 10 min and protected phGluc was extracted using the DNeasy Blood and Tissue kit (Qiagen). The titer of packaged reporter plasmid was determined by TaqMan quantitative PCR (Applied Biosystems) and a standard curve for serial dilutions of phGluc.

For PsV binding studies, labeled PsV content was normalized by Western blotting detection with purified PAB597, a hybridoma supernatant that produces a monoclonal antibody against JCPyV VP1, which we found to cross-react with TSPyV VP1.

### PsV infections

For PsV infection assay cells were seeded to 6,000–7,000 cells per well in a 96-well plate 18 h prior the infection. PsV Infections were performed by adding 5.5 x 10^5^ or 5.5 x 10^6^ PsV particles to each well in 35 μl serum free media for 2 h at 37°C with 30 min agitations. Cells were washed twice with PBS and media was the replaced with phenol red free media with FBS. Mock PsV controls were generated by transfecting 293TT cells with control plasmid and the phGluc reporter plasmid in a 7:1 ratio and then purifying according to the PsV purification protocol. PsV infection was measured after 3 days by detection of secreted luciferase according to the manufacturer’s instructions (BioLux *Gaussia* Luciferase Assay Kit, New England Biolabs) using an opaque 96-well microplate in a GloMax Multi-Detection System Luminometer (Promega) equipped with an autoinjector. Infection assays were done in quadruplicate and repeated three times. The Cell Titer 96 Aqueous Non-Radioactive Cell Proliferation Assay (Promega) was used according to the manufactor’s protocol to test for toxicity after cell treatments and the absorption was measured at 450 nm.

### Treatment of cells with neuraminidase

Prior to flow cytometry experiments, cells were detached non-enzymatically and washed twice with PBS (see above). Then, 10^6^ cells were resuspended in 100 μl PBS and treated with 1 U/ml neuraminidase (*Clostridium perfringens* neuraminidase type V, Sigma-Aldrich) for 30 min at 37°C with agitation every 10 min. Prior to PsV infection or binding, cells were washed once with serum free media for infection studies or PBS for binding studies and incubated with 1 U/ml neuraminidase in PBS or in PBS (Mock) for 30 min at 37°C with agitation every 10 min. Cells were then washed with media and serum free media before PsV infection or twice with PBS before binding studies.

### Treatment of cells with inhibitors of N- and O-linked glycosylations and inhibitor of glycosphingolipid synthesis

Cells were plated in 6-well plates (5 x 10^5^ cells/per well) for the flow cytometry experiment and in a 96-well plate (10^4^ cells/per well) for PsV infections. Glycosylation inhibitors were mixed in media containing 10% FBS at various concentrations to plated cells. Cells were preincubated with tunicamycin (Sigma-Aldrich) in methanol at 37°C for 16–18 h and with BenzylGalNAc (Calbiochem, USA) in DMSO for 48 h prior to the flow cytometry experiment. D,L-threo-phenyl-2-hexadecanoylamino-3-morpholino-propanol (PPMP) (Matreya LLC, USA) in ethanol was added to the media of cells for 6 days, and media supplemented with PPMP was changed after days 2 and 4. Vehicle controls with equivalent volumes of methanol, DMSO or ethanol for the highest concentration were used in each case. The Cell Titer 96 Aqueous Non-Radioactive Cell Proliferation Assay (Promega) was used according to the manufacturer's protocol to assess toxicity of the inhibitors (absorption at 450 nm).

### Crystallization and data collection

TSPyV VP1 was concentrated to 4.8 mg/ml in 20 mM HEPES pH 7.5, 150 mM NaCl, 20 mM dithiothreitol (DTT) and crystallized at 20°C by sitting drop vapor diffusion against a reservoir containing 100 mM sodium malonate pH 5.0, 10% (w/v) PEG 3350. Drops were set up using 1 μl protein solution, 1 μl reservoir solution and 0.2 μl microseeding solution, which was prepared from previously obtained TSPyV VP1 microcrystals. For complex formation with the GM1 glycan and 6’SL, crystals were grown in a drop supplemented with 10 mM GM1 pentasaccharide (Elicityl, France) or 10 mM 6’SL (Carbosynth, UK). Crystals were harvested after five days and transferred stepwise into reservoir solution complemented with the respective glycan and 30% (v/v) glycerol for cryoprotection before flash-freezing them in liquid nitrogen. For complex formation with 3’SL, preformed native TSPyV VP1 crystals were soaked for 15 min in the reservoir solution supplemented with 10 mM 3’SL (Carbosynth, UK) before flash freezing. Data sets were collected at beam lines X06DA and X06SA at the Swiss Light Source (Villigen, Switzerland).

### Structure determination

Diffraction data was processed with XDS [60], and structures were solved by molecular replacement with Phaser in CCP4 [61,62]. The MCPyV VP1 core structure (pdb: 4FMG) was taken as a homology search model for the native TSPyV VP1 structure, which was then used for structure determination of glycan complex structures. Rigid body and simulated annealing refinement was carried out with Phenix [63]. Alternating rounds of model building in Coot [64] and restrained refinement including the translation-libration-screw (TLS) method [65] and 5-fold noncrystallographic symmetry (NCS) restrained refinement were done with Refmac5 [66]. The carbohydrate ligands were located in 2F_o_-F_c_ and F_o_-F_c_ electron density maps. After incorporation into the model, ligands were refined using restraints from the CCP4 library and user defined restraints for the α2,3 and α2,3- glycosidic bonds. The native TSPyV VP1 structure and the structures complexed with 3’SL and 6’SL possess ten VP1 chains in the asymmetric unit, while the asymmetric unit of the VP1-GM1 glycan complex structure comprises five chains. The final VP1 structures contain amino acid residues 33–40, 44–101 and 110–303 for all chains. Residues of the short loop region between the A_new_- and the B-strand (residues 40–44) and the CD-loop (residues 101–110) could be built into defined electron density for some VP1 chains. However, these residues generally display elevated temperature factors, indicating flexibility of these two regions at the bottom of the unassembled VP1 pentamer if not involved in crystal contacts. To assess amino acid conservations on the VP1 surface, an alignment was carried out with Clustal Omega and JalView [67] with the VP1 sequences from OraPyV (NCBI reference YP_003264533) and ApanPyV1 (NCBI reference YP_007195272). Assign values for the conservation of the chemical acid amino acid properties from 10 (conserved; grey) to 0 (non-conserved; red) in JalView were than highlighted on the TSPyV VP1-GM1 complex surface. Values of 2 to 0 are coloured in red.

Structure figures were prepared with PyMOL (The PyMOL Molecular Graphics System, Version 1.3, Schrödinger, LLC).

### Saturation transfer difference (STD) NMR

STD NMR spectra were recorded using 3 mm tubes on a Bruker AVIII-600 MHz spectrometer equipped with a room temperature probe head at 288 K. Data was processed with TOPSPIN 3.0 (Bruker). The sample contained 2 mM GM1 oligosaccharide (Elicityl, France) or 20 μM TSPyV VP1 and 2 mM GM1 oligosaccharide, respectively, in 20 mM K_2_HPO_4_/ KH_2_PO_4_ pH 7.4, 150 mM NaCl, 99%D_2_O. Off- and on-resonance frequencies were -30 ppm and 7.3 ppm, respectively. The irradiation power and length of the selective pulse train was 57 Hz and 2 s, respectively. In order to suppress residual protein resonances a continuous-wave spin-lock pulse with a strength of 3.2 kHz was employed. The relaxation delay was 3 s and a total of 5 k scans were recorded. Spectra were multiplied with a Gaussian window function prior to Fourier transform and referenced to the a-D-Glc anomeric proton as an internal standard [68].

### Accession numbers

Coordinates and structure factor amplitudes were deposited in the RCSB Protein Data Bank (www.pdb.org) under accession codes 4U5Z (unliganded TSPyV VP1), 4U60 (TSPyV VP1-GM1 glycan), 4U61 (TSPyV VP1-6’SL) and 4U62 (TSPyV VP1-3’SL).

## Supporting Information

S1 Fig The binding of Alexa Fluor 488-labelled VP1 pentamers from TSPyV and BKPyV to Mock (PBS) or *Clostridium perfringens* neuraminidase type V pre-treated cells measured by flow cytometry. Non-standardized raw data is shown here for individual experiments. Data was standardized for signals of mock treated cells to obtain the histogram in Fig 1A showing the relative binding of TSPyV VP1 pentamers. The relative average fluorescence from three independent experiments is shown compared to binding to untreated cells. 30,000 gated events were measured for each sample.
(TIF)

S2 Fig Screening cell lines for TSPsV infection. TSPyV pentamers bind to all tested cell lines in a sialic acid-dependent manner (see Fig 1 and S2 Fig panel B), but TSPsV transduction could only be detected for HEK293 and SVGA cells. (A) Common cell lines were infected with TSPsV to obtain information about the tropism. At 72 h post infection the luciferase expression was measured to determine transfection efficiency of TSPsV. Cell lines where TSPsV infection was undetectable are in general able to express the reporter plasmid [40]. The average relative luciferase units (RLUs) for one experiment performed in quadruplet are shown in log scale. Error bars represent standard deviations. Two different dilutions of particles were used and compared to Mock PsV control. The mock PsV control was generated by harvesting HEK293TT cells transfected with control plasmid instead of the capsid expression plasmids and then purifying according to the PsV purification protocol to measure background signal and non-specific transfer of luciferase to infected cells. (B) The binding of Alexa Fluor 488-labelled TSPyV VP1 pentamers to Mock (PBS) or *Clostridium perfringens* neuraminidase type V pre-treated Vero and CHO cells was measured by flow cytometry. BKPyV VP1 pentamers were used as a positive control. Non-standardized raw data is shown here for representative individual experiments. Three independent experiments were performed and 30,000 gated events were measured for each sample.
(TIF)

S3 Fig Mapping the GM1 glycan binding epitope of TSPyV VP1 in solution. Saturation transfer difference (STD) NMR of TSPyV VP1 with the GM1 glycan. From top to bottom: STD-NMR difference spectrum of 50 μM TSPyV VP1 with 1 mM GM1 glycan; ^1^H reference spectrum recorded with the same sample; STD spectrum of the GM1 glycan alone.
(TIF)

S4 Fig Glycolipids are important for TSPsV infection in A549 cells. TSPsV transduction of A549 cells pre-treated with PPMP or the carrier control for 6 days was assayed 72 h post infection by quantification of the secreted luciferase due to transduction of the reporter plasmid phGluc. BKPsV were used as a positive control for a ganglioside-dependent infection. Mock PsV infections were done with a control sample obtained according the PsV purification protocol from cells only transfected with phGluc and control plasmid to assess the background signal of the luciferase assay. PsV experiments were done in triplicate or quintuplicate. The data from the quintuplicate experiment is shown as a representative example. Statistic analysis was performed using the two-tailed unpaired t test.
(TIF)
