# Supplementary figures and images for: Trichodysplasia spinulosa-Associated Polyomavirus Uses a Displaced Binding Site on VP1 to Engage Sialylated Glycolipids
Source: PLoS Pathog. 2015 Aug 24;11(8):e1005112. doi: 10.1371/journal.ppat.1005112 (PMC4547793; doi:10.1371/journal.ppat.1005112)

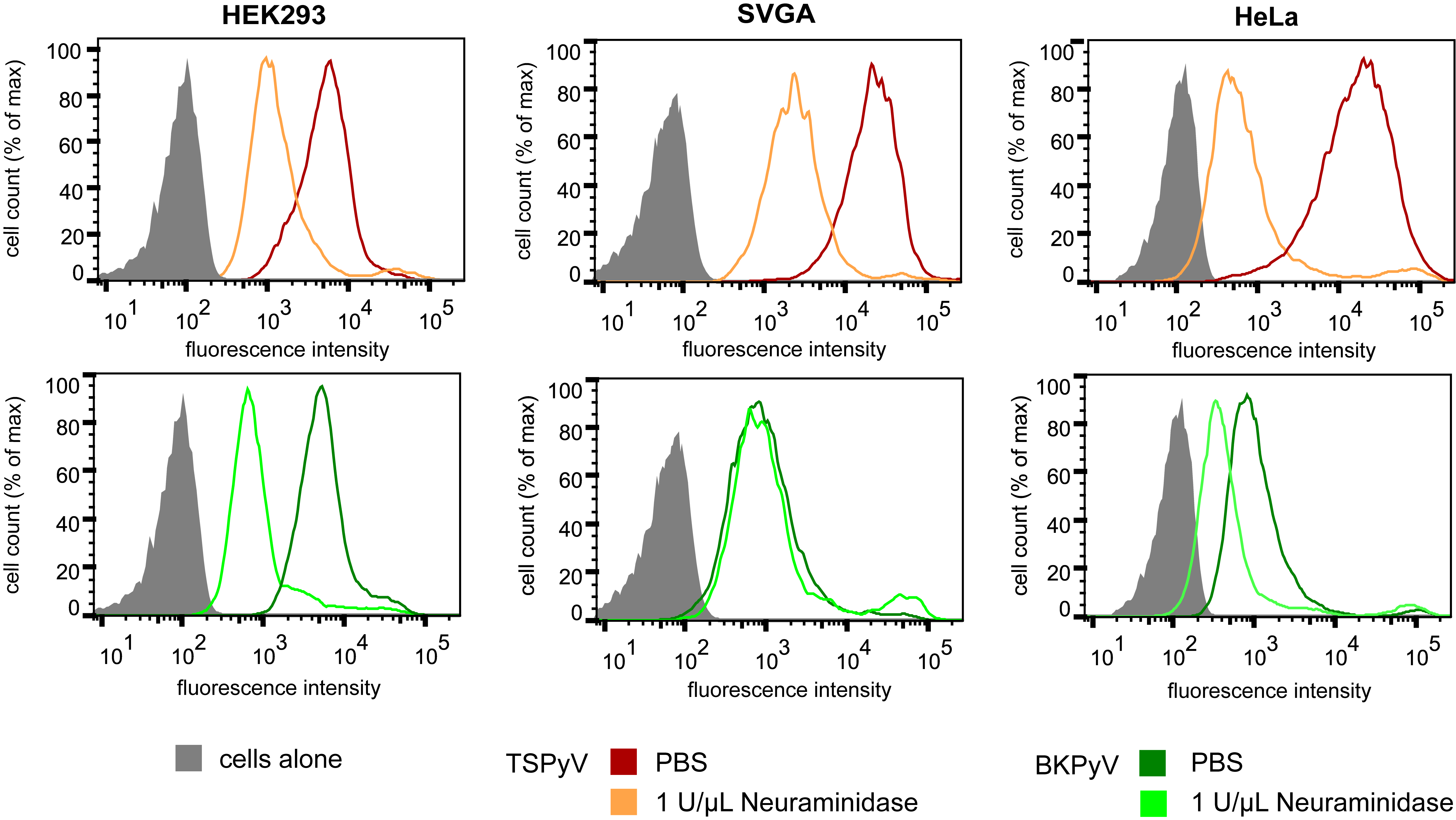

Supplement: S1 Fig — Non-standardized raw data is shown here for individual experiments. Data was standardized for signals of mock treated cells to obtain the histogram in Fig 1A showing the relative binding of TSPyV VP1 pentamers. The relative average fluorescence from three independent experiments is shown compared to binding to untreated cells. 30,000 gated events were measured for each sample. (TIF) [file ppat.1005112.s001.tif]

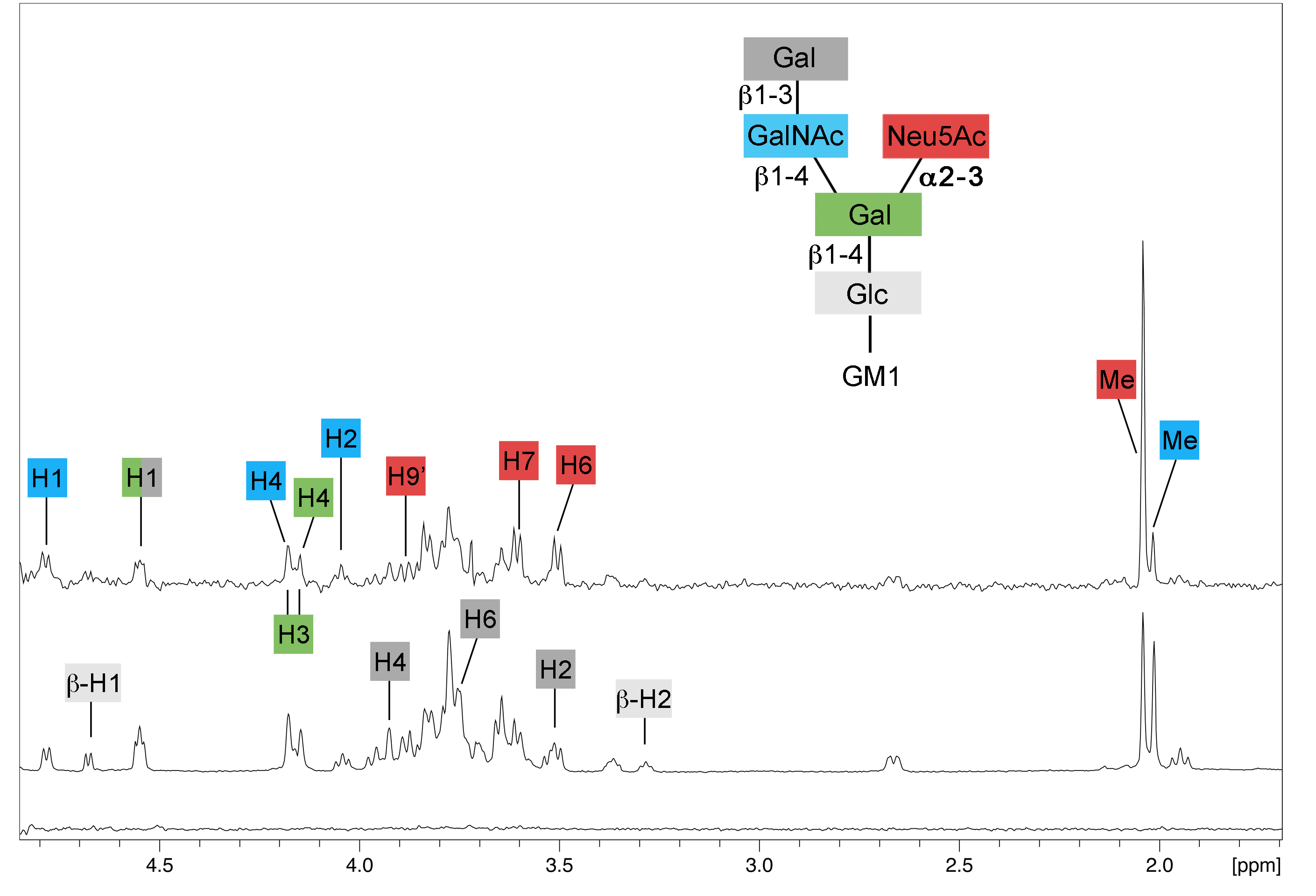

Supplement: S3 Fig — Saturation transfer difference (STD) NMR of TSPyV VP1 with the GM1 glycan. From top to bottom: STD-NMR difference spectrum of 50 μM TSPyV VP1 with 1 mM GM1 glycan; 1H reference spectrum recorded with the same sample; STD spectrum of the GM1 glycan alone. (TIF) [file ppat.1005112.s003.tif]

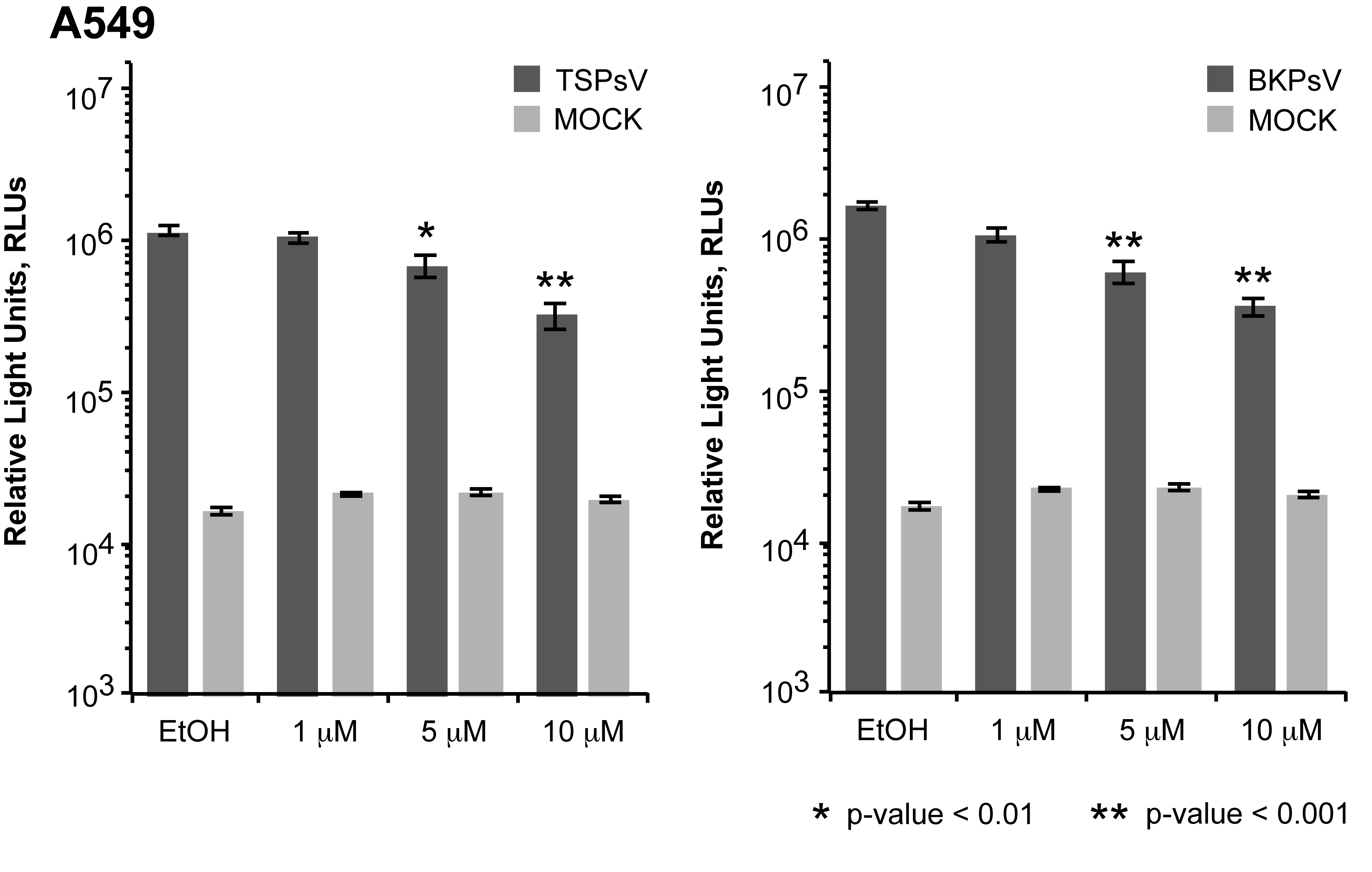

Supplement: S4 Fig — TSPsV transduction of A549 cells pre-treated with PPMP or the carrier control for 6 days was assayed 72 h post infection by quantification of the secreted luciferase due to transduction of the reporter plasmid phGluc. BKPsV were used as a positive control for a ganglioside-dependent infection. Mock PsV infections were done with a control sample obtained according the PsV purification protocol from cells only transfected with phGluc and control plasmid to assess the background signal of the luciferase assay. PsV experiments were done in triplicate or quintuplicate. The data from the quintuplicate experiment is shown as a representative example. Statistic analysis was performed using the two-tailed unpaired t test. (TIF) [file ppat.1005112.s004.tif]
